# A theory-based online health behavior intervention for new university students: study protocol

**DOI:** 10.1186/1471-2458-13-107

**Published:** 2013-02-05

**Authors:** Tracy Epton, Paul Norman, Paschal Sheeran, Peter R Harris, Thomas L Webb, Fabio Ciravegna, Alan Brennan, Petra Meier, Steven A Julious, Declan Naughton, Andrea Petroczi, Aba-Sah Dadzie, Jen Kruger

**Affiliations:** 1 Department of Psychology, University of Sheffield, Western Bank, Sheffield S10 2TP, UK; 2 School of Psychology, University of Sussex, Falmer, BN1 9QH, UK; 3 Department of Computer Science, University of Sheffield, Regent Court, Sheffield, S1 4DA, UK; 4 School of Health and Related Research, University of Sheffield, Regent Court, Sheffield, S1 4DA, UK; 5 School of Life Science, Kingston University, Penrhyn Road, Kingston upon Thames, KT1 2EE, UK

**Keywords:** Randomized controlled trial, Young people, Internet, Self-affirmation, Theory of planned behavior, Implementation intentions, Alcohol, Fruit and vegetables, Exercise, Smoking

## Abstract

**Background:**

Too few young people engage in behaviors that reduce the risk of morbidity and premature mortality, such as eating healthily, being physically active, drinking sensibly and not smoking. The present research developed an online intervention to target these health behaviors during the significant life transition from school to university when health beliefs and behaviors may be more open to change. This paper describes the intervention and the proposed approach to its evaluation.

**Methods/design:**

Potential participants (all undergraduates about to enter the University of Sheffield) will be emailed an online questionnaire two weeks before starting university. On completion of the questionnaire, respondents will be randomly assigned to receive either an online health behavior intervention (*U@Uni*) or a control condition. The intervention employs three behavior change techniques (self-affirmation, theory-based messages, and implementation intentions) to target four heath behaviors (alcohol consumption, physical activity, fruit and vegetable intake, and smoking). Subsequently, all participants will be emailed follow-up questionnaires approximately one and six months after starting university. The questionnaires will assess the four targeted behaviors and associated cognitions (e.g., intentions, self-efficacy) as well as socio-demographic variables, health status, Body Mass Index (BMI), health service use and recreational drug use. A sub-sample of participants will provide a sample of hair to assess changes in biochemical markers of health behavior. A health economic evaluation of the cost effectiveness of the intervention will also be conducted.

**Discussion:**

The findings will provide evidence on the effectiveness of online interventions as well as the potential for intervening during significant life transitions, such as the move from school to university. If successful, the intervention could be employed at other universities to promote healthy behaviors among new undergraduates.

**Trial registration:**

Current Controlled Trials, ISRCTN67684181.

## Background

Eating healthily, being physically active, consuming low levels of alcohol and not smoking are known to reduce the risk of developing serious diseases and conditions such as cancer, heart and circulatory disease, obesity and type 2 diabetes [1]. However, too few young people engage in these health behaviors. For example, the 2008 Health Survey for England [2] revealed that only 20% of young people (aged 16–24) eat five portions of fruit and vegetables per day, less than 50% meet weekly physical activity guidelines, 25% smoke, and 40% exceed daily recommended alcohol limits. In addition to the long-term impact of such behaviors, many health-risk behaviors, such as binge drinking, also contribute to an array of more immediate negative health and social consequences including accidents, injuries, physical violence, sexual assault and poor academic outcomes [3].

Early adulthood is regarded as an exploratory phase with respect to health behaviors [4]; nevertheless, the lifestyle habits that are established during this phase often persist into later life and determine long-term health outcomes [5]. The transition from school to university marks a significant life change in the lives of many young people as studying at university typically involves living away from home for the first time and brings freedom from parental supervision [6]. Importantly, this move also typically breaks the environmental context in which previous unhealthy (or healthy) behaviors were performed and offers young people the opportunity to develop new (healthier) lifestyle habits while at university [7]. Moving to a new location has been found to be involved in 36% of successful attempts to change some aspect of respondents’ lives (e.g., attempts to quit smoking) [8]. However, the change in context can also be problematic, leading to an increase in the performance of health-risk behaviors. For example, binge drinking has been shown to increase during the transition from school to university [9], such that binge drinking is more frequent among university students than among their non-student peers [6].

The present intervention capitalizes on the transition from school to university to promote the adoption of healthy lifestyle habits in young people. There are three main reasons why this transition provides a unique opportunity to intervene in order to promote healthy lifestyle habits. First, it affords the opportunity to target a large proportion of young people in the UK. More than 350,000 students aged 20 or under start university each year, representing around 40% of school leavers [10]. Second, major life transitions, such as the move to university, represent critical or “teachable” moments [11] to intervene in order to promote healthy lifestyle habits. The inherent change in the environmental context, including the disruption of established peer networks, means that peoples’ health beliefs and behaviors are likely to be in a state of flux and therefore more amenable to change. Third, unlike many major life events (e.g., bereavement, loss of employment, divorce) the transition from school to university is a predictable event and one that is experienced by a large proportion of young people in the UK at the same time every year; therefore, interventions can be easily implemented to target a large number of people on an annual basis. The present intervention will be delivered prior to attendance at university in order to exploit this transition, in contrast to previous interventions which have been delivered when students are already at university [12], when health behaviors may be less amenable to change as new peer networks and new habits will have been already formed.

The use of digital technologies holds the potential to deliver interventions designed to promote healthy lifestyle habits to large sections of the population, especially young people, who are prime users of Internet and digital technologies [13,14]. Such interventions are convenient for providers as they are easy to disseminate and low in cost compared to traditional modes of delivery [12]. Furthermore, digital interventions can incorporate interactive materials, such as video streaming and chat rooms, in order to maximize engagement [12]. Digital interventions are also available 24-hours a day and so can be accessed at critical moments [12]. The present intervention will take advantage of widespread access to mobile devices and desktop computing, use of online social media and Internet connectivity, to provide an online space in which participants are encouraged to engage with the intervention material, using methods and platforms with which they are already familiar. A recent meta-analysis confirmed the potential of online health behavior interventions, reporting an overall effect size of *d* = 0.16 on health behavior relative to comparison conditions that did not receive the intervention [12].

### Theoretical bases of the intervention

Evidence indicates that interventions designed to promote health behavior change that are based on theory are more efficacious [12]. The present intervention, therefore, includes three theory-based behavior change techniques to promote healthy lifestyle habits: A self-affirmation task designed to reduce defensive processing of health messages [15], theory-based messages designed to increase people’s motivation to adopt healthy lifestyle habits [16], and implementation intention formation designed to increase the likelihood that good intentions are translated into behavior [17]. The evidence for each of these techniques is considered in turn below.

#### Self-affirmation

Many attempts to promote healthy lifestyle habits fall at the first hurdle because they fail to persuade people that they need to change their behavior, especially those people who are most at risk [15]. Self-affirmation theory hypothesizes that messages about future health risks not only threaten peoples’ physical integrity (e.g., by outlining the future morbidities and heightened risk of premature mortality from continuing risky behavior) but also their sense of being sensible, rational, “adaptively and morally adequate” people (i.e., their “self-integrity”) p. 262, [18]. As a result, people often resist messages about the health risks of certain behaviors (e.g., by derogating the health-risk or counter-arguing) in order to maintain their self-integrity [15].

Self-affirmation – the process of reflecting upon one’s cherished values, actions or attributes – provides a simple and effective technique for reducing defensive resistance to health-risk messages [15]. Encouraging people to self-affirm enables them to feel sufficiently secure about their self-integrity and removes the need to maintain self-integrity by rejecting relevant but unwelcome health-risk information. This, in turn, allows people who have self-affirmed to engage in a more open-minded and balanced appraisal of the health-risk message and its personal relevance. In support of these ideas, self-affirmation has been found to lead to less defensive processing of health-risk information and to positive changes in people’s health-related attitudes, intentions and initial precautionary behavior across a range of health-threats, including those from smoking, alcohol, poor diet and lack of exercise [15]. The proposed intervention will, therefore, encourage participants to self-affirm before being exposed to theory-based messages about the health risks of the target behaviors.

#### Theory-based messages

In order to change health behavior, it is necessary for health messages to target the key motivational factors that underlie such behavior. According to the Theory of Planned Behavior (TPB), the most proximal determinant of behavior is intention which, in turn, is determined by three constructs: (i) attitude (i.e., positive or negative evaluations of performing the behavior), (ii) subjective norm (i.e., perceived social pressure to perform or not perform the behavior), and (iii) perceived behavioral control (i.e., perceived difficulty of performing the behavior) [19]. Underlying each of these constructs are beliefs about (i) the likely consequences of performing the behavior, (ii) the views of specific others and (iii) the power of factors to facilitate or inhibit performance of the behavior, respectively.

The TPB has been used extensively to predict various health behaviors including fruit and vegetable intake, physical activity, alcohol consumption, and smoking. A recent meta-analysis of 237 prospective tests of the TPB in relation to health behavior reported that, on average, the TPB explained 44% of the variance in intention and 19% of the variance in future behavior [20]. The TPB therefore provides a strong theoretical framework for developing interventions to change health behavior [16]. Importantly, the determinants of behavior outlined in the TPB are potentially modifiable, unlike more distal predictors of health behavior, such as gender and ethnicity. Successful interventions based on the TPB have been reported in relation to fruit and vegetable intake, physi-cal activity [21] and heavy alcohol consumption [22]. In an early systematic review of TPB-based interventions, Hardeman et al. [23] reported that such interventions typically had significant, but only small, effects on behavior. However, a more recent meta-analysis found that interventions that changed attitudes, subjective norms or perceived behavioral control had medium effects on intentions and behavior [24]. In order to strengthen the effect of the theory-based messages in the present intervention, extensive formative research was conducted in order to identify and target the specific behavioral, normative and control beliefs that are associated new students’ intentions and behavior in relation to alcohol consumption (binge drinking), physical activity, fruit and vegetable intake and smoking.

#### Implementation intentions

Many people fail to adopt healthy lifestyle habits despite having positive intentions (i.e., strong motivation) to do so. This “intention-behavior gap” is a major obstacle to interventions that seek to promote healthy lifestyle habits, since interventions may increase people’s intentions to change but fail to secure the corresponding change in behavior. The Model of Action Phases [25] distinguishes between motivational processes (those concerned with intention formation) and volitional processes (those concerned with intention realization). Consequently, Gollwitzer [25] differentiates goal intentions (i.e., “I intend to do X”) from implementation intentions, which specify when, where and how a person will act in order to achieve the desired goal (i.e., “If situation Y occurs, then I will initiate goal-directed behavior Z”). An implementation intention takes the form of an if-then plan that links a suitable opportunity to act with a behavioral response that will help people achieve their goal. Forming implementation intentions ensures that the opportunity (specified in the “if” part of the plan) is highly accessible (and so likely to be swiftly and accurately identified) and that the behavioral response (the “then” part of the plan) is performed relatively automatically (i.e., immediately and efficiently) once the critical situation is encountered [26-28]. A meta-analysis of 23 studies found that implementation intentions have a medium-to-large effect on health behaviors (*d* = 0.59) [17], including fruit and vegetable intake, physical activity, alcohol consumption and smoking. Forming implementation intentions is particularly effective for people who already have strong intentions [29]. Thus, implementation intention interventions have been found to be most effective when combined with motivational interventions [30-32].

### The present research

The primary question addressed by the present research is whether an online intervention delivered during the transition from school to university can produce significant changes in the health behaviors (i.e., fruit and vegetable intake, physical activity, alcohol consumption and smoking) of young people. In addition, we will investigate whether the intervention: (i) changes health cognitions (and whether these changes mediate the effect of the intervention on the health behaviors), (ii) enhances health status, (iii) reduces health service usage, (vi) reduces recreational drug use, (v) reduces BMI, and (vi) improves academic performance.

## Method/design

The study protocol was approved by the Department of Psychology Research Ethics Committee at the University of Sheffield (No.: 2012–436).

### Design

A randomized controlled trial will be conducted with two arms: (i) an online intervention targeting four health behaviors during the transition from school to university and (ii) a measurement only control.

### Recruitment, randomization and allocation

All incoming undergraduate students to the University of Sheffield (*N* ≈ 4,500) will be sent an email approximately two weeks before they start university inviting them to take part in the study. Respondents will be provided with a link in the invitation email to a questionnaire which will assess health-related cognitions and behaviors at baseline. Participants will be informed that they will receive £10 for completing all three surveys and that they will be entered into a £100 prize draw after completing each survey.

**Figure 1:**
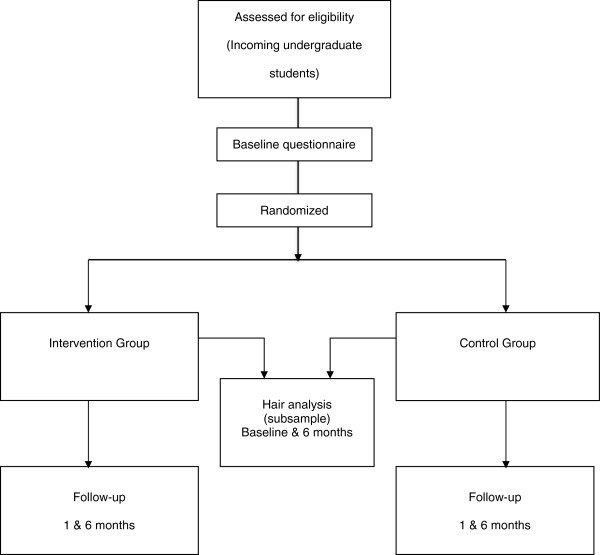
Flowchart of the randomized controlled trial.

The baseline questionnaire will inform respondents that they will be randomly allocated to receive an online resource designed to help them to make healthy choices at university or to a control condition without access to the online resource. Participants will also be asked to give informed consent and agree to allow researchers to access records regarding their academic achievement and university sports facility usage. On completion of the baseline questionnaire, participants will be randomly allocated to the two arms of the trial using a random number generator that is part of the survey software (i.e., SurveyGizmo). Participants in both arms of the trial will be sent emails with links to the follow-up questionnaires one and six months after starting university (see Figure 1). All participants will receive up to three automatic reminders to complete the questionnaires at each time point.

### Intervention

After completing the baseline questionnaire, respondents in the intervention arm of the trial will be directed to the online intervention. On their first visit to the intervention website (following study enrolment information, consent and completion of the baseline questionnaire), participants will be asked to complete a self-affirmation task. Participants will be asked to select their most important personal value from a list of eight commonly held personal values (e.g., sense of humor, academic achievement, relations with family and friends, social skills, spontaneity, artistic skills/aesthetic appreciation, religion/faith/ spirituality, respect/decency/manners) or to provide their own, and to briefly provide a reason why the value is important to them. The resultant information will form part of the user’s profile accessible from the intervention home page that will include brief personal details (e.g., gender, course), hobbies and interests and the participant’s most important personal value (and reason for selecting that value).

After completing their profile, participants will have access to theory-based messages pertaining to each of the four targeted health behaviors. The theory-based messages include a mixture of text and videos, as well as links to other relevant material. The messages were developed on the basis of formative work, in line with TPB guidelines [16], which identified the key behavioral, normative and control beliefs underlying new students’ intentions and behavior for each of the four health behaviors. For each belief, persuasive messages were developed to either support positive beliefs (e.g., binge drinking has a negative effect on your studies) or challenge negative beliefs (e.g., binge drinking helps you to make friends). The content of the messages was informed by further formative research with current university students in order to identify key arguments that support or challenge these beliefs. For example, a key behavioral belief underlying binge drinking among students is that binge drinking is sociable [33] and a good way to make friends at university. Messages were therefore developed to counter this belief (e.g., binge drinking is not the best way to make friends) and to highlight other ways in which students can make friends without binge drinking (e.g., join student clubs and societies). In addition, participants will be able to watch videos of current students talking about health behavior at university that reinforce these messages, as well as follow links to other relevant background material (e.g., information on the effects of binge drinking, lists of socializing opportunities that do not involve alcohol, etc.). In order to ensure that they are not overwhelmed by the volume of material, participants will be able to selectively access information that is of interest to them and opt to access more detailed information (using links to more information and via a search function).

Following exposure to the self-affirmation exercise and theory-based messages, participants will be able to access a planner that contains instructions to form implementation intentions to facilitate the translation of good intentions into action. Participants will be asked to identify (i) a good opportunity to act on their intentions and (ii) a suitable response to their identified opportunity. A series of drop down menus will help participants to identify suitable opportunities to act (e.g., “If I am doing my food shopping…”, “If someone offers me a drink…”) and suitable responses to these opportunities (e.g., “…then I will look out for special offers on fruit and vegetables”, “…then I will tell them that I have work to do tomorrow”). Suggestions were based on formative research with current students. Moreover, participants will also be able to generate their own opportunities and responses. Once participants have identified an opportunity to promote a healthy lifestyle and a suitable response, they will be prompted to link them in the “If [opportunity], then [response]” format that defines successful implementation intention interventions [34]. Participants will also be instructed to repeat their plan to themselves several times to ensure that they can remember their plan. The plans that participants make will be stored in a ‘plan repository’ that can be reviewed at any time with a direct link from the home page. If they wish, participants can also set a reminder for each plan to be repeated at a set time interval.

### Implementation

Two weeks before they start university, potential participants will be sent an email inviting them to participate in the study. Those who complete the baseline questionnaire and are randomized to the intervention condition will be directed to the intervention which will be delivered via a web-based interface that is accessible from laptops and desktop computers, with a scaled-down mobile version designed to support interactive use on smaller, more resource-limited devices (e.g., smartphones or tablets). The intervention also includes a dedicated Twitter feed, Facebook and Google+ pages that will be used to highlight some of the intervention materials (e.g., videos) and to provide facts relating to the targeted beliefs to encourage engagement with the intervention. Participants will also have the option to store information (from *U@Uni* or other sources) in their private space (*Me@Uni),* and to share this information with other participants. Immediately before the start of the second university semester, participants in the intervention condition will be sent an email informing them that an Android smartphone application (app) is available to download from the *U@Uni* website. The app will allow users to access the intervention on the go. Synchronization with changes made on other devices or using the web-based version will occur where there is network access. It is anticipated that the release of the mobile app will promote greater engagement with the intervention as well as acting as a reminder to use the intervention at another transition period (i.e., when returning to university after the Christmas vacation at the start of an exam period).

### Measures

Unless otherwise indicated, the primary and secondary measures will be completed at baseline (two weeks before starting university), and approximately one month and six months after starting university.

#### Primary outcome measures

Health behavior will be measured using a mixture of reliable and validated measures including items from the Health Survey for England (HSE; an annual survey of health and health behavior in England) [2] and the General Lifestyle Survey (GLF; an annual survey of health behavior in the UK) [35]. These measures will facilitate health economic modeling of the data from the trial alongside epidemiological data available from HSE and GLF.

##### Fruit and vegetable intake

Fruit and vegetable intake (portions per day) will be assessed with items based on the HSE. Participants will be asked to think about the preceding day and indicate if they ate nine different types of fruit and vegetables (e.g., pulses, raw vegetables) and how much of each type they ate (e.g., “Did you eat any salad yesterday?” and “How many cereal bowls of salad did you eat yesterday?”).

##### Physical activity

The International Physical Activity Questionnaire (IPAQ) will be used to assess levels of physical activity. Respondents will be asked to indicate how many times, and for how long, they have engaged in vigorous exercise (i.e., defined as “activities that take hard physical effort and make you breathe much harder than normal”), moderate exercise (i.e., defined as “activities that take moderate physical effort and make you breathe somewhat harder than normal”) and walking in the past 7 days. Responses will be computed into METs (metabolic equivalent of task) to provide a total IPAQ score. An additional question will ask about sedentary activity. The IPAQ has undergone extensive reliability and validity testing across 12 countries [36]. The IPAQ items will be supplemented by more detailed questions on walking [2].

##### Alcohol

Alcohol consumption will be assessed using items from the GLF to provide a measure of units of alcohol per week and number of binge sessions per week (e.g., participants will be asked to indicate on which days they have drunk alcohol in the last 7 days and the type and amount of alcohol they drank on each of those days). The Alcohol Use Disorders Identification Test (AUDIT) [37] will also be used at the six-month follow-up to assess hazardous and harmful patterns of alcohol use at university.

##### Smoking

Items based on the HSE will be used to assess participants’ current smoking status and the typical number of cigarettes/amount of tobacco smoked.

#### Secondary outcome measures

**Social cognitive variables** Brief measures of social cognitive variables for each behavior will be constructed in line with current recommendations [16]. Measures of self-efficacy (e.g., “If I wanted, I could easily engage in regular exercise at university”), perceived control (e.g., “Whether or not I engage in regular exercise at university is under my control”) and intention (e.g., “I intend to engage in regular exercise at university”) will be taken at all time points. Measures of attitude (e.g., “Engaging in regular exercise at university would be… good/bad”), subjective norms (e.g., “Most people who are important to me think I should/should not engage in regular exercise at university) and planning (e.g., “To what extent do you have a detailed plan about how to engage in regular exercise at university?”) will be taken at the one and six month follow-ups.

##### Health status

The EQ-5D-3L [38] is a short, standardized measure of health status that assesses levels of severity (no problems/some or moderate problems/extreme problems) in five dimensions: mobility, self-care, usual activities, pain/discomfort and anxiety/depression. The measure provides a descriptive profile and a single index value for health status, and is recommended as the measure of health-related quality of life for health economic evaluations in the UK [39].

##### Recreational drug use

To estimate the prevalence of recreational drug use in the sample, respondents will be asked to indicate the number of “yes” answers (0 or 5, 1, 2, 3, 4) to five questions – four of which have a 50% population prevalence (e.g., odd or even date of birth) and one of which is on their use of recreational drugs. In this way it is possible to estimate the prevalence of recreational drug use in the sample without being able to identify whether individual participants do or not use recreational drugs. This way of asking about drug use has been shown to encourage accurate reporting of a behavior that is illegal and could be regarded as socially undesirable [40].

##### BMI

Participants will record their height and weight so that BMI can be calculated.

##### Health services usage

Self-report data on the use of the health service (e.g., GP visits, hospitalizations) will be gathered at the six-month follow-up to explore whether there are differences in healthcare resource use between the intervention and control arms.

##### Academic performance

Average exam marks and registration status (i.e., registered, transferred, withdrawn, leave of absence) will be used to assess academic performance at the end of each semester. These data will be gathered from university records (with the participants’ consent).

##### Use of university sports facilities

Data on use (membership, number of visits) of the university sports facilities will be collected from Sport Sheffield (with the participants’ consent).

##### Engagement with the digital intervention

Various measures of engagement with the intervention will be recorded such as the overall number of visits, the number and type of pages visited, the number of implementation intention plans completed.

##### Biochemical measures

A random sample of approximately 100 participants from each arm of the trial will be recruited by email to assess biochemical markers of health behavior when they start university and at six-month follow-up. At each time point, participants will provide a hair sample (3cm long) that will be liquefied and analyzed for biochemical markers of various health behaviors related to alcohol consumption, fruit and vegetable intake, cigarette smoking and recreational drug use. Following extraction procedures, markers of alcohol, vitamins and minerals and nicotine will be quantified using liquid chromatography with tandem mass spectrometric detection (LC-MS/MS). In addition, evidence for social drug use will be detected by screening for commonly used drugs and their metabolites. A 6430 triple quadruple mass spectrometer (Agilent Technologies UK) will be employed with a dynamic-multiple reaction monitoring-liquid chromatography mass spectrometry (DYN-MRM-LC-MS/MS) method. These participants will also have their height and weight measured to calculate BMI.

### Results

#### Sample size calculations

A previous Internet-based intervention study on alcohol use among US students [41] sent emails to all new freshmen at the start of the semester; 55% completed the baseline questionnaire, of whom 63% were followed-up at one month. For the proposed trial, we have assumed that 50% of participants will complete the baseline questionnaire, of whom 60% will be followed-up at six months, producing a total sample size of 1200 (i.e., 600 per arm of the trial). With 600 participants per arm, the trial will have at least 80% power to detect a small effect size of *d* = 0.20 [42] with a two tailed significance level of .0127 (the p value has been adjusted to allow for multiplicity in the co-primary endpoints). For the hair analysis, participants will be randomly sampled from each arm of the trial with a maximum of 100 per arm which will be sufficient to detect a small to medium effect size of *d* = 0.40 (alpha = .05, power =. 80) [42].

#### Analysis

Primary analyses will adopt an intention-to-treat approach (i.e., all participants randomized to the intervention group will be analyzed regardless of whether they actually accessed the intervention). This approach provides a more conservative estimate of the effectiveness of an intervention than comparing the control condition with those in the intervention group who engaged with the intervention rather than the full group [43]. The primary outcomes will be performance of the four health behaviors (i.e., fruit and vegetable intake, physical activity, alcohol consumption, smoking) at the six-month follow-up. The primary analysis will comprise a series of analyses of covariance (ANCOVAs) to assess the impact of the intervention on performance of the targeted health behaviors at six months, controlling for baseline health behavior scores. For smoking, the analysis will assess the proportion of smokers at six-month follow-up in each arm of the trial with appropriate adjustment of baseline smoking status using a logistic regression. Statistical significance will be declared if any of the primary endpoints are significant at .0127. The analysis will be repeated for the same endpoints at one month as a secondary analysis.

Secondary analyses will assess changes in social cognitive variables for each health behavior, over time, by trial group. Mediation analyses [44] will test whether changes in social cognitive variables mediate the effects of the intervention on the targeted health behaviors. Moderation analyses, using ANCOVA, will test whether the intervention is more effective for certain demographic sub-groups (e.g., based on deprivation index scores, ethnicity, etc.) and whether engagement with the intervention moderates the effectiveness of the intervention on health behaviors. Other analysis data sets will be considered as supporting analyses such as the per protocol and the as treated. The above analyses will be repeated for the various secondary outcome measures under consideration (e.g., biochemical markers, health status, academic performance).

#### Additional analyses

**Randomization check** There will be an exploratory analysis of participants in the two arms of the trial at baseline to ensure that the randomization was successful. Baseline differences between conditions will be controlled for in relevant analyses.

##### Comparison of drop-outs versus completers

In addition, there will be an assessment of differential drop-out rate in the two arms of the trial using survival analysis methods (e.g., Kaplan-Meier plots); additionally, differences between *drop-outs* and *completers* on baseline measures will be assessed.

##### Process evaluation

A process evaluation will be conducted to assess participants’ reactions to the intervention and possible contamination effects. Interviews will be conducted with 20 randomly selected participants from the intervention condition in order to determine the acceptability of, and level of engagement with, the online materials. An online survey at the end of the trial (after the six-month follow-up) with a random selection of 100 control participants will explicitly ask about potential contamination (e.g., did the participant talk to other students about the trial, did they access the *U@Uni* resources, did they change anything they did as a result of finding out about the intervention resources, etc.).

##### Health economic evaluation

The planned health economic analysis will assess the cost-effectiveness of the proposed intervention, balancing the health benefits (in terms of Quality Adjusted Life Years - QALYs) achieved against the costs. Standard approaches to costing the development and implementation of the intervention will be undertaken, separating the fixed costs of set up and implementation from the variable costs per targeted recipient. The full costs of developing the intervention will be estimated as well as the likely costs of adapting the intervention to other universities.

Short-term direct effects on health-related quality of life (as measured by the EQ-5D-3L during the trial) will be compared to intervention costs and short-term health service usage costs in order to estimate the cost per QALY gained within the trial period. Long-term cost-effectiveness modelling will be used to estimate the medium and longer-term impact of changes in behavioral risk-factors (fruit and vegetable intake, physical activity, alcohol consumption and smoking) on costs and QALYs. An integrated model of the consequences of these behavioral risk factor changes on long-term cost and QALY outcomes will be developed based on review of existing models, published literature, and data from the HSE and GLF. The health economic evaluation will consider the extent to which behavior and risk factor changes following the intervention may be sustained or not sustained over the medium and long term.

## Discussion

This paper described (i) an online theory-based intervention (*U@Uni*) that has been designed to promote healthy lifestyle habits in young adults as they make the transition from school to university, and (ii) the randomized controlled trial that will assess its effectiveness.

The intervention has several strengths regarding its timing, delivery and content. First, the intervention capitalizes on the transition from school to university – a significant life change during which health beliefs and behavior may be more malleable. Previous research has indicated that changes in location are important in breaking established habits [7,8] and therefore provide a critical moment to intervene. Moreover, given that over 350,000 students start university each year in the UK, the intervention has the potential to impact on the health behavior habits of a large proportion of young people. Second, the intervention will be delivered using digital technologies, of which young people are the prime users [13,14], thereby ensuring that the intervention is both convenient and engaging to use. Third, the intervention incorporates three complementary behavior change techniques, each of which has a strong theory and evidence base; namely, (i) self-affirmation [15], (ii) theory-based messages [16], and (iii) implementation intentions [17]. A recent meta-analysis reported that online health behavior interventions that were based on theory and used a combination of behavior change techniques produced stronger effect sizes [12].

The proposed research is of theoretical importance as, to date, no studies have examined the efficacy of combining self-affirmation, theory-based messages and implementation intentions to change health behavior. For example, previous studies examining the effects of self-affirmation on health behavior have been limited in the choice of variables to target and the development of the health messages has typically lacked a strong theoretical basis [45]. In addition, while self-affirmation manipulations have been found to lead to positive intentions and initial changes in health behavior, there is limited evidence that they promote sustained behavior change [15]. As a result, additional volitional techniques, such as implementation intentions [25], may be required to support behavior change. In line with this argument, studies have shown that interventions that supplement theory-based health messages with implementation intentions produce larger effects [30-32].

It is likely that the combination of the three behavior change techniques in the online intervention will have a synergistic effect as, together, they address three key factors that may hinder attempts to change health behavior. First, the self-affirmation task will reduce defensive processing and thereby increase engagement with the health messages. Second, the theory-based messages will ensure that the key beliefs underlying each health behavior are targeted. Third, asking participants to form implementation intentions will assist the translation of good intentions into behavior. If the intervention is found to improve students’ health behavior during the first six months of university life, future experimental work may be required to identify the “active ingredients” of the intervention, for example, whether it is necessary to include the self-affirmation manipulation and/or prompt participants to form implementation intentions along with the health messages.

The results of the proposed study will also be of practical importance as the intervention has the potential to improve the health behaviors (and health status) of a large proportion of the population of young people as they enter university. If successful, the intervention could be adapted to other universities in the UK and tested in a cluster RCT. To further test the generalizability of the intervention it may also be applied in other countries where large numbers of young people enter university. However, it should be noted that in many European countries, as well as in Australia and the USA, it is not normative to move away from one’s home city (and one’s home) to go to university, and many students may start university before the legal age for drinking alcohol which may impact on the intervention's suitability. Finally, the intervention could also be adapted and delivered to other school leavers including those who enter the work environment (especially when this involves a move away from the home environment).

The proposed study also has a number of potential limitations. First, there is only a limited time period in which to recruit potential participants as the announcement of ‘A’ level results on which university places in the UK are contingent only happens a few weeks before the start of term. There is also likely to be some attrition over the course of the proposed study. In order to maximize recruitment and retention, a number of strategies that have been identified to increase response rates to online surveys [46] will be used, including non-monetary incentives (i.e., gift vouchers), deadlines for responses, an offer of a summary of the study findings, and a statement that others have responded (in reminder emails).

Second, the time before starting university and the first few months at university are likely to be hectic and stressful for many students which may serve to reduce engagement with the intervention. However, we have taken a number of steps to try to minimize this problem. In particular, the nature of the intervention (i.e., its relevance to the significant life transition) is likely to increase interest in, and engagement with, the intervention. In addition, the use of digital technologies will ensure that the intervention can be easily accessed 24 hours a day. Nonetheless, participants’ engagement with the intervention (e.g., number of times the intervention is accessed) will be measured and its potential moderation of the effectiveness of the intervention will be tested. Furthermore, the introduction of the smartphone application immediately prior to the second semester will hopefully further prompt engagement with the intervention as it coincides with the move back to university after the Christmas vacation and exam period.

Third, given that the trial is being conducted in a single university, there is a possibility of contamination between the intervention and control groups (whereby control participants learn about the intervention and use it themselves). While there are advantages to conducting the trial at a single university (namely, increased control over extraneous factors like the availability of healthy food, opportunities to binge drink, etc.), potential contamination effects will be assessed by conducting a brief online survey at the end of the trial with a random selection of 100 participants in the control group to explicitly ask about potential contamination.

Fourth, the intervention targets multiple health behaviors. Webb et al. (2010) [12] reported that Internet-based interventions that targeted multiple health behaviors tended to report lower effect sizes (*d* = 0.12) than those that targeted single behaviors (*d* = 0.17). The reduced effectiveness of interventions that target multiple health behaviors could be due to a dilution effect (e.g., whereby participants only engage with material for one of the health behaviors) or the effect of compensatory health beliefs (e.g., whereby the performance of an unhealthy behavior such as binge drinking is believed to be negated by performing a healthy behavior such as exercise) [47]. However, should the intervention result in small changes across the four health behaviors, when combined, these changes may still impact on future health outcomes.

## Conclusion

This paper describes the protocol for a randomized controlled trial designed to evaluate the effectiveness of an online theory-based intervention (*U@Uni*) that targets four health behaviors in young people during transition from school to university. Self-report and objective measures of health behavior, health outcomes and academic performance will be taken. The proposed study will also include a health economic evaluation to weigh the short and long term health benefits against the costs of the intervention. The results of the trial will therefore provide further data on the effectiveness of theory-based, online health behavior interventions and their potential to impact on long-term health outcomes.

## Abbreviations

ANCOVA: Analysis of covariance; AUDIT: Alcohol use disorder identification test; BMI: Body mass index; GLF: General lifestyle survey; HSE: Health survey for England; IPAQ: International physical activity questionnaire; MANOVA: Multivariate analysis of variance; MET: Metabolic of equivalent task; QALY: Quality adjusted life years; TPB: Theory of planned behavior.

## Competing interests

The authors declare that they have no competing interests.

## Authors’ contributions

TE wrote the first draft of this paper and finalized the paper after feedback from all other authors. PN, PS, PH, TW, FC, PM, AB, SJ, DN and AP designed and wrote the original proposal. PN, PS, PH, TW and TE were primarily responsible for the design of the intervention content, FC and AD for the design of the website/app, PM, AB and JK for the health economics evaluation, SJ for the statistical analysis plan, AP for the indirect prevalence estimation of drug use, and AP and DN for the biochemical markers aspect of the study. All authors commented on drafts and approved the final version of the paper.

## Pre-publication history

The pre-publication history for this paper can be accessed here:

http://www.biomedcentral.com/1471-2458/13/107/prepub
